# Ultrasmall Fe_2_O_3_ Tubular Nanomotors: The First Example of Swarming Photocatalytic Nanomotors Operating in High-Electrolyte Media

**DOI:** 10.3390/nano13081370

**Published:** 2023-04-14

**Authors:** Lingxia Yu, Manyi Yang, Jianguo Guan, Fangzhi Mou

**Affiliations:** State Key Laboratory of Advanced Technology for Materials Synthesis and Processing, International School of Materials Science and Engineering, Wuhan University of Technology, 122 Luoshi Road, Wuhan 430070, China; xiaoyuer531@163.com (L.Y.); yangmanyi@whut.edu.cn (M.Y.); guanjg@whut.edu.cn (J.G.)

**Keywords:** nanomotors, phototaxis, collective behaviors, ion tolerance, photocatalysis

## Abstract

Self-propelled chemical micro/nanomotors (MNMs) have demonstrated considerable potential in targeted drug delivery, (bio)sensing, and environmental remediation due to their autonomous nature and possible intelligent self-targeting behaviors (e.g., chemotaxis and phototaxis). However, these MNMs are commonly limited by their primary propulsion mechanisms of self-electrophoresis and electrolyte self-diffusiophoresis, making them prone to quenching in high electrolyte environments. Thus, the swarming behaviors of chemical MNMs in high-electrolyte media remain underexplored, despite their potential to enable the execution of complex tasks in high-electrolyte biological media or natural waters. In this study, we develop ultrasmall tubular nanomotors that exhibit ion-tolerant propulsions and collective behaviors. Upon vertical upward UV irradiation, the ultrasmall Fe_2_O_3_ tubular nanomotors (Fe_2_O_3_ TNMs) demonstrate positive superdiffusive photogravitaxis and can further self-organize into nanoclusters near the substrate in a reversible manner. After self-organization, the Fe_2_O_3_ TNMs exhibit a pronounced emergent behavior, allowing them to switch from random superdiffusions to ballistic motions near the substrate. Even at a high electrolyte concentration (*C_e_*), the ultrasmall Fe_2_O_3_ TNMs retain a relatively thick electrical double layer (EDL) compared to their size, and the electroosmotic slip flow in their EDL is strong enough to propel them and induce phoretic interactions among them. As a result, the nanomotors can rapidly concentrate near the substrate and then gather into motile nanoclusters in high-electrolyte environments. This work opens a gate for designing swarming ion-tolerant chemical nanomotors and may expedite their applications in biomedicine and environmental remediation.

## 1. Introduction

Micro/nanomotors (MNMs) are tiny machines that can produce autonomous motions powered by chemical fuels or by external energies, including light, magnetic, electric or acoustic fields [1,2,3,4]. To date, researchers have developed various MNMs with bio-friendly materials, powerful thrust, and controllable motions [5,6,7]. Meanwhile, similar to flocking birds and shoaling fish in nature, they may further self-organize into micromotors purely through local diffusiophoretic, electrostatic, magnetic and hydrodynamic interactions, and show intriguing collective behaviors that single individuals do not have, such as enhanced driving forces, strong robustness, and adaptive reconfigurations [8,9,10,11,12]. Owing to their unique mobility, different proof-of-concept demonstrations of possible applications of these MNMs have been reported, including cargo transport [13,14], sensing [15,16], microsurgery [17,18], wound healing [19], and environmental remediation [3,20].

Chemically-driven MNMs are always of particular interest in the field because of their autonomous nature and possible intelligent self-targeting tactics and behaviors (e.g., chemotaxis and phototaxis) [21,22,23]. However, the majority of chemical MNMs rely on self-electrophoresis or electrolyte diffusiophoresis, suffering from a fundamental limitation: motion quenching in high-electrolyte solutions (e.g., body fluids and natural waters) [24,25]. This is because their self-propulsion is dominated by the solid-liquid interaction between the charged particles and the electric double layer, which would be shielded at high ion concentrations according to the classical Helmholtz-Smoluchowski theory [25]. To address the low ion tolerance of chemically driven MNMs, two major strategies have been developed, including surface modification and porous structural design. For example, Tang et al. proposed a polyelectrolyte-coating strategy to increase the Dukhin number of light-driven MNMs to enhance their self-electrophoretic propulsion in electrolyte solutions [24]. In addition, Sitti et al. reported that proper porosity can effectively circumvent the limitations of ion accumulation around the particles and demonstrated that porous C_3_N_4_ microparticles could operate in high-ion-concentration media utilizing the internal flow of ions and liquids in their textural and structural pores [26]. However, these two strategies necessitate the implementation of specific designs in terms of surface properties and internal structures, thereby limiting their applicability. More importantly, the swarming chemical MNMs with high ion tolerance remain underexplored, despite their great potential to execute complex tasks in high-electrolyte biological media or natural waters.

Herein, we demonstrate ultrasmall photocatalytic Fe_2_O_3_ tubular nanomotors (Fe_2_O_3_ TNMs) and their light-driven propulsions and swarming behaviors in high-electrolyte media. Due to the asymmetric photocatalytic reactions on the irradiated and shadowed surfaces, as well as intrinsic asymmetry in the inner cavity, the Fe_2_O_3_ TNMs show superdiffusive photogravitactic behaviors based on self-electrophoresis upon vertical upward UV or visible light irradiation This allows them to move downward when away from the substrate and then undergo random superdiffusive motions upon reaching the substrate. Thus, the Fe_2_O_3_ TNMs can rapidly accumulate near the substrate and, owing to local inter-motor phoretic interactions, further gather into nanoclusters. This allows them to switch motion behaviors from random superdiffusions to ballistic motions. The light-triggered clustering behavior is reversible and can be spatially regulated by adjusting the shape and size of the light spot. As the Fe_2_O_3_ TNMs have a relatively thick electrical double layer (EDL thickness *κ*) compared to their radius (*a*) in high-electrolyte media, they show high ion tolerance in their self-electrophoretic propulsions and phoretic-interaction-induced clustering behaviors. Thus, they have a high *EI50* value (30.2 mM), which is a measure of the ionic concentration at which the speed of the microswimmers is reduced by 50% [24]. The Fe_2_O_3_ TNMs also show apparent collective clustering behaviors in the medium, even with a high electrolyte (NaCl) concentration (*C_e_*) of 150 mM (comparable to that in body fluids). A new parameter of *SEI50*, which is defined as the ionic concentration at which the cluster size of swarming nanomotors is reduced by 50%, was also proposed to evaluate their ion tolerant swarming behaviors. The *SEI50* of the swarming Fe_2_O_3_ TNMs is 28.4 mM, which is near their speed-related *EI50*. This parameter may greatly facilitate the evaluation of the ion tolerance of those nanomotors that are difficult to be tracked at an individual level under an optical microscope. This work opens a gateway for designing swarming, ion-tolerant chemical nanomotors. The developed ultrasmall Fe_2_O_3_ TNMs may serve as promising candidates for future environmental and biomedical applications due to their high ion tolerance.

## 2. Materials and Methods

### 2.1. Synthesis of Fe_2_O_3_ TNMs

The Fe_2_O_3_ TNMs were fabricated via a hydrothermal method. First, α-Fe_2_O_3_ nanotubes were synthesized by a hydrothermal method described by Jia et al. [27]. Briefly, FeCl_3_, NaH_2_PO_4_, and Na_2_SO_4_ were dissolved in 80 mL distilled water to form a homogeneous solution with a concentration of 0.02, 5 × 10^−4^, and 5.5 × 10^−4^ mol/L, respectively. The solution was transferred to a Teflon-lined stainless-steel autoclave for hydrothermal treatment at 220 °C for 48 h. The α-Fe_2_O_3_ nanotubes were obtained after washing the precipitate with distilled water and absolute ethanol, and drying it in a vacuum at 60 °C. Then, a 3 mL acetone suspension of the α-Fe_2_O_3_ nanotubes (100 mg) was loaded into a porcelain boat and put into a tube furnace. The tube furnace was subjected to air removal through being fed a N_2_ flow for 10 min. Afterward, the sample was calcined at 400 °C for 2 h under continuous N_2_ flow. Finally, the sample was annealed in the air for another 2 h at 400 °C in the tube furnace to obtain Fe_2_O_3_ TNMs with mixed α and γ phases.

### 2.2. Characterization

Scanning electron microscopy (SEM) images were obtained using a Hitachi S-4800 field-emission SEM (Hitachi Co. Ltd., Tokyo, Japan). The transmission electron microscopy (TEM) images were captured by a JEM-2100F TEM (JEOL, Tokyo, Japan). A powder X-ray diffraction (XRD) analysis of the sample was conducted on the D8 Advanced X-ray Diffraction Meter (l1/41.5418 Å, Bruker, Karlsruhe, Germany). The zeta potential of the sample was measured by a NanoBrook 90 Plus Zeta analyzer (Brookhaven Instruments, Holtsville, NY, USA).

### 2.3. Light-Driven Propulsions and Swarming Behaviors of Fe_2_O_3_ TNMs

A 10 μL aqueous suspension containing Fe_2_O_3_ TNMs (1 mg/mL), tetramethylammonium hydroxide (TMAH) (2.5 mM)(RHAWN Chemical Reagent Co., Ltd., Shanghai, China) and H_2_O_2_ fuel was placed on a glass slide. Then, the light from the built-in Leica EL6000 light source of an inverted optical microscope (Leica DMI 3000B, Wetzlar, Germany) was used to actuate the Fe_2_O_3_ TNMs in the aqueous suspension. The motions and clustering behaviors of the Fe_2_O_3_ TNMs were investigated at different on/off states, intensities (*I*), wavelengths and light-spot shapes, as well as at different concentrations of the H_2_O_2_ fuel (*C*_f_), respectively. All videos were analyzed using Video Spot Tracker V08.01 software. Video Spot Tracker software was used to analyze average speed and mean square displacement (MSD) of the Fe_2_O_3_ TNMs. To calculate the cluster size, a quantitative analysis was performed using ImageJ software.

### 2.4. Photocatalytic Degradation of Rhodamine 6G

A mixture of 0.5 mL of Rhodamine 6G (0.01 mM), 0.5 mL of Fe_2_O_3_ particle suspension (0.2 mg/mL), 0.5 mL of H_2_O_2_ solution (10 wt.%), and 0.5 mL of deionized water was added into a 5 mL centrifuge tube. Then, a UV-LED light source with a maximum *I* of 1800 mW/cm^2^ was placed under the centrifuge tube. Before UV exposure, Rhodamine 6G was adsorbed by the nanomotors for 15 min to ensure adsorption equilibrium. After UV exposure for a certain time, the concentration of Rhodamine 6G in the solution was analyzed using a UV−visible spectrophotometer (UV-2550, Shimadzu, Japan). The analysis was conducted immediately after separating the nanomotors via centrifugation at 10,000 rpm for 3 min.

## 3. Results and Discussion

### 3.1. Conceptual Design of Ion-Tolerant Fe_2_O_3_ TNMs

**Figure 1:**
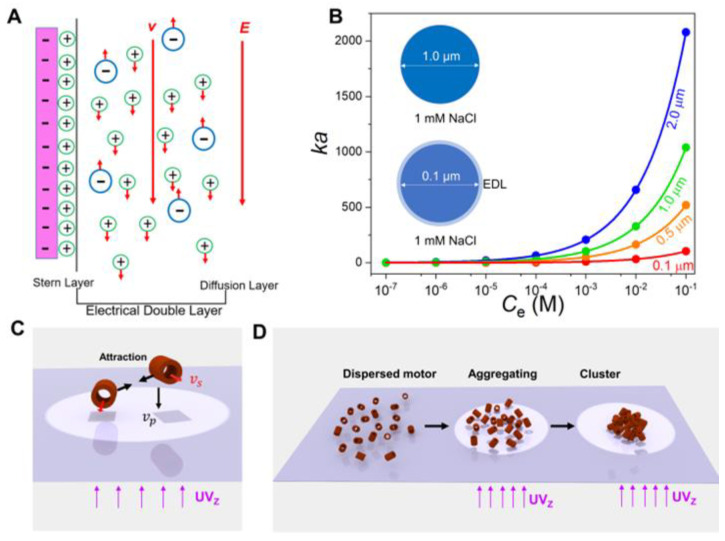
Conceptual design of ion-tolerable ultrasmall Fe_2_O_3_ TNMs. (**A**) Schematic diagram of the electrical double layer on a micro/nanomotor based on self-electrophoresis or electrolyte diffusiophoresis. (**B**) the ratio of the radius (*a*) to the EDL thickness (*1/κ*) of a micro/nanomotor (*κa*) as a function of ion concentrations (*C_e_*). (**C**) Light-driven propulsions and local interactions of two adjacent nanomotors under vertical upward UV irradiation (UV_Z_). (**D**) Schematic diagram of swarming behaviors of the Fe_2_O_3_ TNMs under UV_Z_ irradiation.

With self-electrolysis and electrolyte self-diffusiophoresis, a micro/nanomotor can generate a local electric field (*E)* around it via reaction surface flux. This local *E* then exerts a body force on the free charge in the motor’s EDL to generate surface electroosmotic slip, causing the motor to move in the opposite direction of the slip (Figure 1A) [25]. The micro-sized self-diffusiophoretic and self-electrophoretic motors have a particularly low *EI50* number of less than 0.1 mM [26]. Thus, they would decelerate rapidly with the increasing *C_e_* in the medium and usually became completely quenched at *C_e_* less than 5 mM. The ion quenching of the micro/nanomotor fundamentally relies on the collapsing of the EDL in the electrolyte solution [28]. Small nanomotors would have a relatively high EDL thickness (Debye length, 1/*κ*) compared to the motor radius (*a*), as characterized by the dimensionless parameter *κa* (Equation (1)) [29].

(1) $$\kappa a=a\sqrt{4\pi l_{B}C_{e}}\;$$

Here, $l_{B}$ ($l_{B}=q^{2}/\varepsilon k_{B}T$) is the Bjerrum length, representing the distance at which the interaction energy between two ions with ion charge *q* in a dielectric medium with dielectric constant *ε* equals the thermal energy unit $k_{B}T$, where $k_{B}$ is the Boltzmann constant and *T* is the temperature. For the nanomotors in pure water, κa≪1. As the *C_e_* increases in the medium, the *κa* increases sharply, indicating the thinning of the micro/nanomotors’ EDL. However, the small motor shows a slower thinning trend (i.e., *κa* increase). This means that the smaller motor would have a relatively thicker Debye length (1/*κ*) compared to its *a*, strengthening its self-electrophoretic or diffusiophoretic propulsion. For instance, in a NaCl solution with a *C_e_* of 1 mM, the EDL thickness ($\kappa^{-1}$ = 9.62 nm) of a micromotor (*a* = 1 μm) is only ~1% compared to its *a* (nearly invisible in the upper inset in Figure 1B), and its self-electrophoretic propulsion would be severely slowed. In stark contrast, a nanomotor with a size of 100 nm has an EDL of about ~10% of its *a* (light blue ring in the lower inset in Figure 1B). Thus, it is expected that small self-electrophoretic and self-diffusiophoretic nanomotors would have a high ion tolerance.

To implement this prediction, we designed an ultrasmall Fe_2_O_3_ TNM driven by photocatalytic reactions. As Fe_2_O_3_ is a semiconductor material with a band gap of 2.2 eV [30], electron-hole pairs can be formed in Fe_2_O_3_ under light excitation, and further participate in photocatalytic reactions of H_2_O_2_ (Equations (2)–(4)) [31,32,33,34].

(2) $${\mathrm{Fe}}_{2}O_{3}+h\nu \to h^{+}+e^{-}\;$$

(3) $$H_{2}O_{2}+2h^{+}\to O_{2}+2H^{+}\;$$

(4) $$H_{2}O_{2}+2e^{-}+2H^{+}\to 2H_{2}O\;$$

It is known that photogenerated holes in Fe_2_O_3_ have a much shorter diffusion length (2–4 nm) compared to that of the electrons (tens of micrometers), and thus they tend to stay on the illuminated side to participate in photocatalytic H_2_O_2_ oxidation. According to the condition of electrical neutrality, the photocatalytic H_2_O_2_ oxidation (Equation (3)) prevails and releases H^+^ at the illuminated side of the Fe_2_O_3_ TNM. Consequently, the photocatalytic H_2_O_2_ reduction dominates and consumes H^+^ (Equation (4)) at the shadowed side. These asymmetric photocatalytic reactions produce a [H^+^] gradient across the Fe_2_O_3_ nanomotor, and generate a local electric field (*E*) pointing from the illuminated side to the shadowed side [35,36]. This local *E* then induces a phoretic slip on the negatively-charged Fe_2_O_3_ TNMs, and causes them to move toward the incident light (in positive phototaxis, *v*_p_) based on self-electrophoresis [37]. In addition, due to the inevitable asymmetry of the inner cavity, the Fe_2_O_3_ TNMs may also perform random superdiffusions (*v*_s_) in the plane, as they normally would when exposed to light (Figure 1C) [38]. Furthermore, the self-electrophoretic slips would produce a local flow field and induce phoretic interactions between adjacent motors (Figure 1C). Thus, as the local density of the nanomotors (*C_n_*) rises, swarming behaviors are also expected to emerge (Figure 1D). More specifically, once the light irradiation begins, the dispersed nanomotors gradually aggregate with their neighbors based on local phoretic interactions, and then form into large clusters with clearer distinguishability (Figure 1D). As predicted earlier, the designed Fe_2_O_3_ TNMs are expected to still work in high-electrolyte media due to their ultrasmall size, leading to ion-tolerable self-electrophoretic propulsions and collective behaviors. Furthermore, as the swarming behaviors of the nanomotors are directly governed by their self-electrophoresis and motion speed, the swarming capability and the cluster size may also be exploited as new parameters to evaluate the ion tolerance of the nanomotors. This could especially be the case for those nanomotors that are difficult to track under an optical microscope at an individual level.

### 3.2. Characterization and Propulsions of Fe_2_O_3_ TNMs

**Figure 2:**
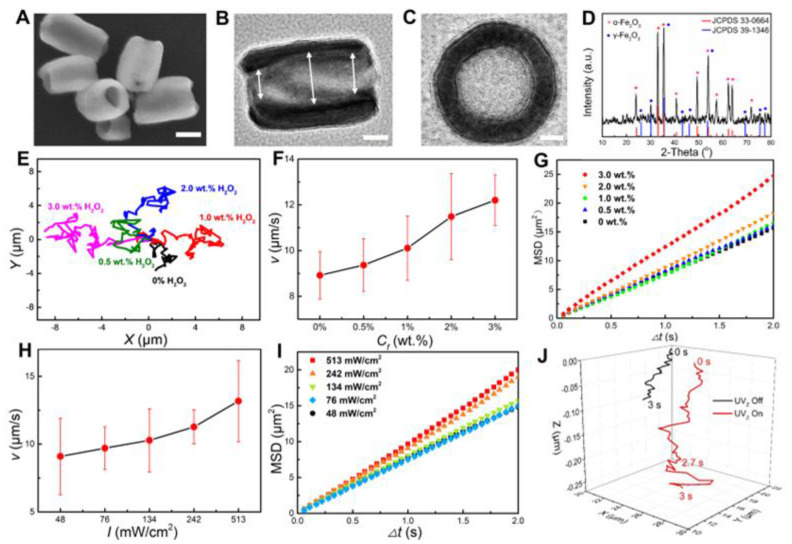
Structural characterization and motion behaviors of the Fe_2_O_3_ TNMs. (**A**) SEM and (**B**,**C**) TEM images of the Fe_2_O_3_ TNMs. Scale bars in (**A**–**C**) are 100 nm, 50 nm, and 25 nm, respectively. (**D**) XRD pattern of the Fe_2_O_3_ TNMs. (**E**) Trajectories of the Fe_2_O_3_ TNMs within 5 s under UV_Z_ irradiation at different fuel concentration (*C*_f_). (**F**) Speed (*v*) of the Fe_2_O_3_ TNMs as a function of C_f_. The light intensity (*I*) is kept at 513 mW/cm^2^. (**G**) Average MSD of the Fe_2_O_3_ TNMs versus the time interval (Δ*t*) at different C_f_. (**H**) The *v* of the nanomotors at different *I*. The *C*_f_ is kept at 3.0 wt.%. (**I**) MSD as a function of Δ*t* at different *I*. (**J**) Three-dimensional trajectories of two typical Fe_2_O_3_ TNMs when UV_Z_ is on and off.

The Fe_2_O_3_ TNMs were fabricated by elaborately calcining hydrothermally-obtained α-Fe_2_O_3_ nanotubes (Figure S1) alternately in reductive and oxidative atmospheres (see details in Materials and Methods). The obtained sample had a reddish-brown color (Figure S2), and SEM observation revealed that the nanomotors had a diameter of 140 nm and an estimated average length of 230 nm (Figure 2A). The tubular structure of the nanomotors was confirmed by TEM images (Figure 2B,C), which showed that they had an inner diameter of 80 nm and a tube shell thickness of 30 nm. Slight asymmetry in the inner cavity was frequently observed, as confirmed by the slight size difference of the two opening ends (Figure 2B). This asymmetry facilitated their in-plane self-propulsions. Motivated by the enhanced separation and transfer of the photogenerated charges at α/γ phase junctions [39], we elaborately controlled the crystalline phase of the nanomotors. As confirmed by an X-ray diffraction (XRD) analysis, the Fe_2_O_3_ TNMs have well mixed α- and γ- phases (Figure 2D). A zeta potential test revealed that the Fe_2_O_3_ TNMs have a negative surface charge of −37.4 mV (Figure S3). The UV-vis absorbance spectrum of the Fe_2_O_3_ TNMs revealed that they have a broad light absorption range of 200 to 800 nm (Figure S4).

When irradiated by a vertical upward UV light (UV_Z_) with an intensity (*I*) of 513 mW/cm^2^, the Fe_2_O_3_ TNMs settled near the substrate displayed superdiffusive motions in the aqueous medium, with 3.0 wt.% H_2_O_2_ with a speed (*v*) of 12.5 µm/s. The motion trajectory of the Fe_2_O_3_ TNMs is shown in Figure 2E. Conversely, in the absence of H_2_O_2_, the Fe_2_O_3_ TNMs exhibited only weak random walks and a much slower velocity (8.9 μm/s), suggesting that the nanomotors were fueled by H_2_O_2_. The photocatalytic activity of the Fe_2_O_3_ TNMs was verified earlier by their capability for photocatalytic degradation of Rhodamine 6G in the medium (Figure S5). Thus, the light-driven propulsion of the Fe_2_O_3_ TNMs is a result of the photocatalytic degradation of the H_2_O_2_ fuel. As the *C*_f_ increased, the *v* of the Fe_2_O_3_ TNMs increased accordingly, as demonstrated in Figure 2F. The superdiffusion of the nanomotors was systematically analyzed using mean square displacement (MSD) (Figure 2G). This analysis allowed us to determine the long-term translational diffusion coefficient (*D*) by extracting the slope of the MSD plot under both fueled and unfueled conditions according to MSD = 4*D*Δ*t* [40,41]. When the nanomotors were unfueled, the *D* was found to be 1.6 μm^2^/s, which closely approximates the translational diffusion coefficient (*D_t_*) calculated from the Stokes-Einstein equation *D_t_ = k_B_T*/(6*πηa*), where *η* is the viscosity of the solution [42]. In contrast, when the Fe_2_O_3_ TNMs were irradiated by UV_Z_ and fueled with 3.0 wt.% H_2_O_2_, the *D* was found to be 3.1 μm^2^/s, representing an approximate 94% increase compared to the unfueled condition.

The speed of Fe_2_O_3_ TNMs can also be regulated by adjusting the light intensity (*I)* of the UV_Z_ light. As the photon flux is directly proportional to *I*, increasing *I* generates more electron-hole pairs in the Fe_2_O_3_ TNMs for photocatalytic reactions, resulting in increased propulsion. Figure 2H illustrates that at a fixed H_2_O_2_ concentration of 3.0 wt.%, the speed of the nanomotors increased from 9.0 to 12.5 µm/s as the *I* increased from 48 to 513 mW/cm^2^. We noted that Fe_2_O_3_ TNMs show a higher speed than pure α-Fe_2_O_3_ nanomotors (Figure S6), due to the enhanced separation and transfer of the photogenerated charges at α/γ phase junctions [39]. Similarly, we observed an increase in the average MSD slope with increasing *I* (Figure 2I). The apparent *D* of the Fe_2_O_3_ TNMs at 48 mW/cm^2^ was determined to be 1.8 μm^2^/s, and it increased by about 72% when *I* increased to 513 mW/cm^2^ (*D* = 3.1 μm^2^/s).

Of particular interest to us was that the Fe_2_O_3_ TNMs suspended in the bulk aqueous phase exhibited intriguing positive photogravitaxis. In the absence of UV_Z_ light, the Fe_2_O_3_ TNMs suspended in the bulk aqueous phase (away from the substrate) only exhibit a limited displacement in the direction of gravity. In sharp contrast, upon exposure to UV_Z_ light irradiated vertically upward from the microscope lens, the Fe_2_O_3_ TNMs displayed rapid movement in the direction of the light and gradually approached the microscope focus, as illustrated in Figure 2J and Video S1. The photogravitactic velocity *v*_p_ of the Fe_2_O_3_ TNMs was determined to be about 0.1 µm/s. Upon landing on the substrate, the Fe_2_O_3_ TNMs exhibit superdiffusive motion (as evidenced by the red trajectory near the substrate in Figure 2J) similar to their counterparts previously settled near the substrate (Figure 2E). Thus, they demonstrated a three-dimensional superdiffusive photogravitaxis under UV_Z_ illumination.

### 3.3. Swarming Behaviors of Fe_2_O_3_ TNMs

**Figure 3:**
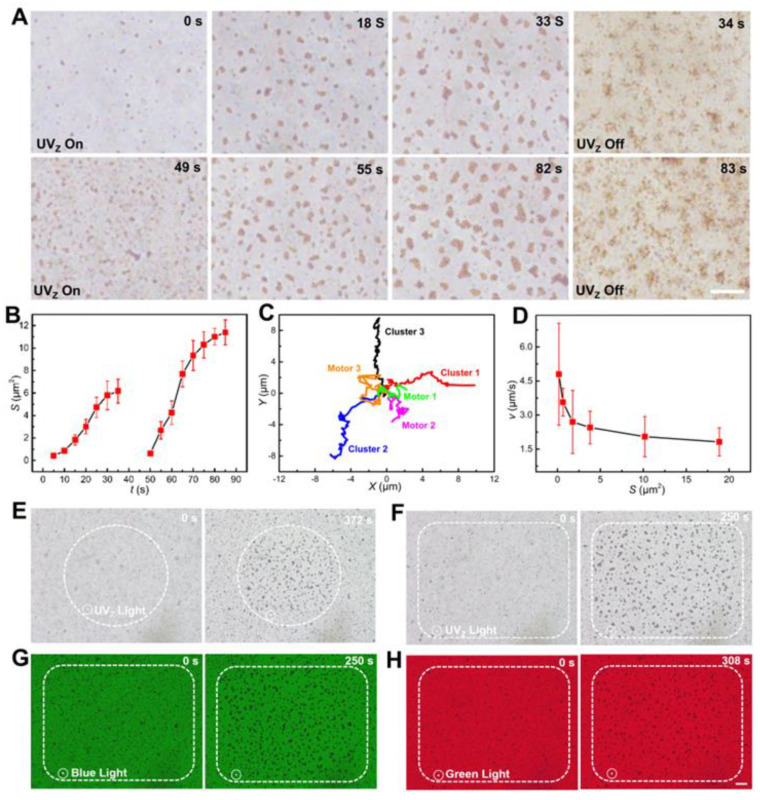
Swarming behaviors of the Fe_2_O_3_ TNMs. (**A**) Time-lapse microscopic images of the Fe_2_O_3_ TNMs depicting their reversible clustering behaviors in response to UV_Z_ irradiation (*I* = 513 mW/cm^2^). The scale bar is 10 μm. (**B**) Corresponding changes of the cluster size (*S*) over time. (**C**) Motion trajectories of three typical single nanomotors and three nanomotor clusters. (**D**) The *v* of the clusters with different *S*. (**E**,**F**) Clustering of the nanomotors in a circular (**E**) and rectangular (**F**) UV_Z_ light spot, respectively. (**G**,**H**) Clustering of the nanomotors in a rectangular spot of blue (480 nm) and green light (538 nm), respectively. The scale bar is 20 μm.

Similar to swarming behaviors observed in living organisms such as flocking birds and shoaling fish [43,44], the Fe_2_O_3_ TNMs exhibit intriguing collective behaviors (Figure 3A and Video S2). Under UV_Z_ irradiation, the superdiffusive photogravitaxis of the suspended Fe_2_O_3_ TNMs enables them to sediment near the substrate, resulting in a rapid increase in their local number density (0–18 s in Figure 3A). Subsequently, the nanomotors aggregate into small clusters, which, grow into larger clusters over time due to the continuous photogravitactic sedimentation of dispersed nanomotors and the merging of small adjacent clusters (18–33 s in Figure 3A). Upon turning off the UV_Z_ light, the formed clusters immediately disassemble into dispersed nanomotors (33–34 s in Figure 3A). Once the UV_Z_ light is reintroduced (49 s in Figure 3A), the Fe_2_O_3_ TNMs reorganize into clusters (49–82 s in Figure 3A) and further dissociate when the UV_Z_ light is turned off again (82–83 s in Figure 3A). The reversible assembly-disassembly behaviors can be explained by the induced attractive phoretic interactions among the Fe_2_O_3_ TNMs under UV_Z_ irradiation, as well as the short-range electrostatic repulsion that impedes their agglomeration after the cessation of light exposure. We believe that the neither the possible heat effects from the motor’s photothermal conversion nor the light sources contribute to the clustering of the Fe_2_O_3_ TNMs, as evidenced by the fact that they show no obvious clustering behaviors when fuel is not present in the medium (Figure S7).

Next, we performed a statistical analysis to examine the impact of vertical UV_Z_ irradiation on the size (*S*) of nanomotor clusters in two on-off cycles, as depicted in Figure 3B. During the first cycle, the average *S* of the clusters increased to 6.2 μm^2^ within 35 s of continued UV_Z_ radiation. We then allowed the formed clusters to disassemble into dispersed nanomotors by ceasing the UV_Z_ radiation for 15 s in preparation for the second clustering cycle. In the second cycle, we observed a faster growth rate of the clusters, with an average *S* of 11.4 μm^2^ achieved within the same duration. This result can be attributed to the significantly increased local number density of the nanomotors after the first cycle, which led to a shorter superdiffusion length for them to aggregate with neighbors, thereby facilitating the re-clustering of the nanomotors through phoretic interactions. The maximum *S* of the formed clusters is ultimately limited by the concentration of the Fe_2_O_3_ TNMs, as they tend to eventually sediment near the substrate and then aggregate with neighbors there.

One pronounced emergent behavior was observed after the dispersed Fe_2_O_3_ TNMs organized into clusters. As shown in Figure 3C, compared to superdiffusive single Fe_2_O_3_ TNMs, the clustered Fe_2_O_3_ TNMs demonstrate ballistic motion due to their significantly reduced Brownian rotations. Furthermore, single Fe_2_O_3_ TNMs demonstrate diffusive trajectories (Motor 1-3 in Figure 3C), while nanomotor clusters demonstrate ballistic trajectories (Cluster 1-3 in Figure 3C). On the other hand, as the size *S* increased, the clusters showed a decreasing *v* due to the increased resistance from the viscous fluid and the reduction of shape asymmetry. As shown in Figure 3D, the speed of the cluster decreased gradually from 4.7 to 1.8 μm/s as the *S* increased from 0.159 to 18.857 μm^2^.

As the clustering behavior occurs only when the Fe_2_O_3_ TNMs are exposed to UV_Z_ radiation, the clustering of the Fe_2_O_3_ TNMs can be spatially controlled. To verify this spatial control, we adjusted the size and shape of the UV_Z_ light spot by setting apertures in the optical microscope. We observed that the Fe_2_O_3_ TNMs within the circular UV_Z_ light spot gradually aggregated into clusters, while those outside the spot remained dispersed (Figure 3E). Similarly, we observed a rectangular clustering zone when a rectangular UV_Z_ light spot was created (Figure 3F). Additionally, owing to the narrow bandgap of 2.2 eV exhibited by Fe_2_O_3_, visible-light irradiation can also induce light-shaping clustering behavior in Fe_2_O_3_ TNMs. Although it was difficult to distinguish and analyze the light-triggered propulsions of individual Fe_2_O_3_ TNMs under visible-light irradiation, pronounced clustering of the nanomotors was observed in a rectangular spot of blue (480 nm, 1.1 W/cm^2^) and green light (538 nm, 1.5 W/cm^2^) (Figure 3G,H). Figures S8 and S9 summarize the various cluster patterns formed in circular and rectangular spots of UV, blue, and green light, respectively (see also Video S3).

### 3.4. Ion Tolerance of Fe_2_O_3_ TNMs

**Figure 4:**
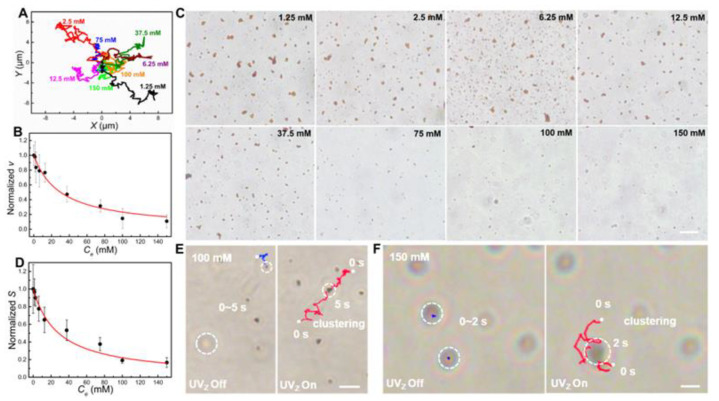
Ion tolerance of the Fe_2_O_3_ TNMs. (**A**) Trajectories of the light-driven Fe_2_O_3_ TNMs at different *C*_e_. (**B**) The normalized speed *v* of the nanomotors versus *C*_e_. (**C**) Microscopic images describing the clusters of the Fe_2_O_3_ TNMs after irradiated by UV_Z_ irradiation for 30 s. The scale bar is 10 μm. The *I* of the applied UV_Z_ light in these experiments is 513 mW/cm^2^. (**D**) The normalized *S* of the nanomotor clusters versus *C*_e_. (**E**,**F**) Microscopic images showing light-activation and clustering of the Fe_2_O_3_ TNMs stuck on the substrate at *C*_e_ of 100 (**E**) and 150 mM (**F**), respectively. Scale bars in (**E**,**F**) are 4 μm, 2 μm.

Although various light-driven MNMs based on photocatalytic semiconductors (e.g., TiO_2_, ZnO, and Fe_2_O_3_) have been developed, their functionality is mainly limited to aqueous media with low ion concentrations, as they are relatively large and are propelled through mechanisms of electrolyte self-diffusiophoresis or self-electrophoresis [38,45]. In contrast, as predicted in Figure 1, the developed Fe_2_O_3_ TNMs may exhibit ion-tolerant phoretic motions due to their ultrasmall size. To investigate the ion tolerance of the Fe_2_O_3_ TNMs, we monitored their motion under the UV_Z_ irradiation (*I* = 513 mW/cm^2^) in aqueous media with varying *C_e_*. As the *C_e_* increased from 1.25 to 150 mM, the trajectory of the Fe_2_O_3_ TNMs shortened over the same 30 s interval, indicating weakened self-electrophoresis (Figure 4A). The weakened self-electrophoresis of the nanomotors was also confirmed through an MSD analysis, suggesting a decrease in diffusivity (*D*) with increasing *C_e_* (Figure S10). Nevertheless, the nanomotors exhibited an *EI50* value as high as 30.2 mM (Figure 4B), indicating that their speed was reduced by only 50% at a high *C_e_* of 30.2 mM. This value is 7.6 times higher than that of reported polyelectrolyte-coated ion tolerant photocatalytic nanomotors [24], revealing the high ion tolerance of the developed ultrasmall Fe_2_O_3_ TNMs.

Due to the high ion tolerance, the ultrasmall Fe_2_O_3_ TNMs also show pronounced swarming behaviors in high-electrolyte solutions (Figure 4C and Video S4). When the *C*_e_ was lower than 75 mM, the formed nanomotor clusters could be easily distinguished under UV_Z_ irradiation for 30 s. When the *C*_e_ reached 100 and 150 mM, a marked decrease in the number and size of nanomotor clusters was observed (Figure 4C). After careful statistical analysis, we found that the clusters’ size *S* increases over time under UV_Z_ light at all *C_e_* levels, but the rate of increase in *S* diminishes with the *C*_e_ (Figure S11). Even at high *C_e_* levels of 100 and 150 mM, a clear growth in *S* of the clusters within a 30 s duration of UV_Z_ irradiation can still be observed, compared to the same without UV_Z_ illumination (Figure S11A). As shown in Figure 4D, the *S* of the formed clusters decreases with the increasing *C_e_*, similar to the effect of *C_e_* on *v*. Thus, we propose a new parameter, *SEI50*, which represents the ionic concentration at which the cluster size *S* of swarming nanomotors is reduced by 50%, to evaluate their ion tolerant swarming behaviors. The *SEI50* of the swarming Fe_2_O_3_ TNMs is determined to be 28.4 mM, which is near speed-related *EI50* (30.2 mM). The *SEI50* parameter may greatly facilitate the evaluation of the ion tolerance of those nanomotors that are difficult to track at an individual level under an optical microscope. The formed clusters could still move in high-electrolyte media and their speed was found to decrease with the increasing *C*_e_, as verified by the clear ballistic motions of a typical cluster in the medium with a *C_e_* of 37.5 mM (Video S5) and statistical results of their speed at different *C*_e_ (Figure S11B). When the *C_e_* increased to 100 and 150 mM, many Fe_2_O_3_ TNMs tended to stick to the glass substrate (Fe_2_O_3_ TNMs with short blue trajectories in Figure 4E,F) because of the largely reduced motor-substrate electrostatic repulsion However, the reduced repulsion could facilitate the observation of their light activation. As shown Video S6, the static motors stuck on the substrate are activated instantly once the UV_Z_ is on, and then aggregate with their neighbors promptly, as verified by the red trajectories in Figure 4E,F. This result further confirms that the ultrasmall Fe_2_O_3_ TNMs still show noticeable light-driven propulsions and swarming behaviors at a high *C*_e_ of up to 150 mM. As predicted in the conceptual design (Figure 1), the high ion tolerance is mainly attributed to the ultrasmall size of the Fe_2_O_3_ TNMs, as confirmed by the fact that the solid Fe_2_O_3_ nanomotors with a similar size (Figure S12A) also have high ion tolerance in their clustering behaviors (Figure S12B). On the other hand, their tubular structure makes them show strong superdiffusion under light irradiation, as verified by their higher *D* than that of solid nanomotors (e.g., *D* = 2.6 and 1.6 μm^2^/s at *C*_e_ of 37.5 mM for tubular and solid nanomotors, respectively) derived from the MSD curves (Figure S12C).

## 4. Conclusions

In summary, we have developed ultrasmall photocatalytic tubular nanomotors and demonstrated their ion-tolerant superdiffusive photogravitaxis and collective behaviors. The ultrasmall Fe_2_O_3_ TNMs with mixed α and γ phases are fabricated large scale by a hydrothermal method, the Fe_2_O_3_ TNMs show superdiffusive photogravitactic behaviors based on self-electrophoresis upon vertical upward UV or visible light irradiation. This allows them to move downward when away from the substrate and then undergo random superdiffusive motions upon reaching the substrate. These behaviors result in rapid crowding of the nanomotors near the substrate, which further leads to their collective clustering facilitated by local inter-motor phoretic interactions. Notably, the light-triggered clustering behavior is reversible, allowing the Fe_2_O_3_ TNMs to switch between random superdiffusions and ballistic motions in response to the light irradiation. Moreover, the clustering behavior can be spatially regulated by adjusting the shape and size of the light spot. Due to the thick EDL relative to its size (small *κa*) in high-electrolyte media, the ultrasmall Fe_2_O_3_ TNMs show high ion tolerance, with an *EI*50 value as high as 30.2 mM in their self-electrophoretic propulsions. Consequently, the Fe_2_O_3_ TNMs show apparent collective clustering behaviors with a *SEI50* of 28.4 mM in the medium, even with a high *C_e_* of 150 mM. Future research directions may include surface modification and functionalization of the Fe_2_O_3_ TNMs to enhance their compatibility with biological environments and living organisms, and to test their competence in performing various tasks. Together with these advances and their ion-tolerant propulsions, the ultrasmall Fe_2_O_3_ TNMs serve as promising candidates for environmental and biomedical applications, such as pollutant degradation in natural waters and in-vitro cell sorting and biomarker enrichment in microfluidic devices, as well as in-vivo motile-targeting drug delivery and photo-(chemo-)therapy within the body.

## Data Availability

The datasets generated during and/or analyzed in the current study are available from the corresponding author on reasonable request.

## Supplementary Materials

The following supporting information can be downloaded at: https://www.mdpi.com/article/10.3390/nano13081370/s1, Figure S1: SEM images of α-Fe_2_O_3_ nanotubes obtained from the hydrothermal procedure; Figure S2: The digital photograph of Fe_2_O_3_ TNMs; Figure S3: Zeta potential of the Fe_2_O_3_ TNMs; Figure S4: The UV-vis absorbance spectrum of the Fe_2_O_3_ TNMs; Figure S5: Absorbance spectra of Rhodamine 6G in the medium with the Fe_2_O_3_ TNMs before and after UV exposure (1.8 W/cm^−2^) for different times; Figure S6: Speed (*v*) of the α-Fe_2_O_3_ nanotubes as a function of *C*_f_; Figure S7: Time-lapse microscopic images of the Fe_2_O_3_ TNMs in pure water under UV_Z_ irradiation (*I* = 513 mW/cm^2^); Figure S8: Clustering behaviors of the Fe_2_O_3_ TNMs in a circular spot of UV_Z_ (360 nm), blue (480 nm) and green light (538 nm), respectively; Figure S9: Clustering behaviors of the Fe_2_O_3_ TNMs in a rectangular spot of UV_Z_ (360 nm), blue (480 nm) and green light (538 nm), respectively; Figure S10: Average MSD of the Fe_2_O_3_ TNMs at different *C_e_* versus the time interval (Δ*t*); Figure S11: The increment of *S* over time (*t*) in the medium with different *C_e_*, and the speed *v* of the formed clusters at different *C_e_*; Figure S12: Characterization and ion-tolerant motion behaviors of solid Fe_2_O_3_ nanomotors. Video S1: Superdiffusive downward photogravitactic motions of the Fe_2_O_3_ TNMs in response to UV_Z_ irradiation; Video S2: Reversible clustering behaviors of the Fe_2_O_3_ TNMs in response to UV_Z_ irradiation (*I* = 513 mW/cm^2^); Video S3: Clustering behaviors of the Fe_2_O_3_ TNMs under lights with different wavelengths (light colors) and light-spot shapes; Video S4: Clustering behaviors of the Fe_2_O_3_ TNMs at different *C_e_* under UV_Z_ irradiation for 30 s; Video S5: Translational motions of a Fe_2_O_3_ TNM cluster in the aqueous medium with *C_e_* of 37.5 mM; Video S6: Light-activation and clustering behaviors of the Fe_2_O_3_ TNMs stuck on the substrate at *C_e_* of 100 mM and 150 mM at high magnification, respectively; Video S7: Clustering behaviors of solid Fe_2_O_3_ nanomotors at different *C_e_* under UV_Z_ irradiation for 30 s.
