# Supplementary material for: Ultrasmall Fe2O3 Tubular Nanomotors: The First Example of Swarming Photocatalytic Nanomotors Operating in High-Electrolyte Media
Source: Nanomaterials (Basel). 2023 Apr 14;13(8):1370. doi: 10.3390/nano13081370 (PMC10143400; doi:10.3390/nano13081370)
Supplement: Supplementary file 1 [file nanomaterials-13-01370-s001.zip › nanomaterials-2332959 Supplementary Materials.pdf]

nanomaterials

Supplementary Materials

Ultrasmall Fe_2O_3 Tubular Nanomotors: The First Example of Swarming Photocatalytic Nanomotors Operating in High−Electrolyte Media

Lingxia Yu, Manyi Yang, Jianguo Guan and Fangzhi Mou *

State Key Laboratory of Advanced Technology for Materials Synthesis and Processing, International School of Materials Science and Engineering, Wuhan University of Technology, 122 Luoshi Road, Wuhan 430070, China; xiaoyuer531@163.com (L.Y.); yangmanyi@whut.edu.cn (M.Y.); guanjg@whut.edu.cn (J.G.)

* Correspondence: moufz@whut.edu.cn

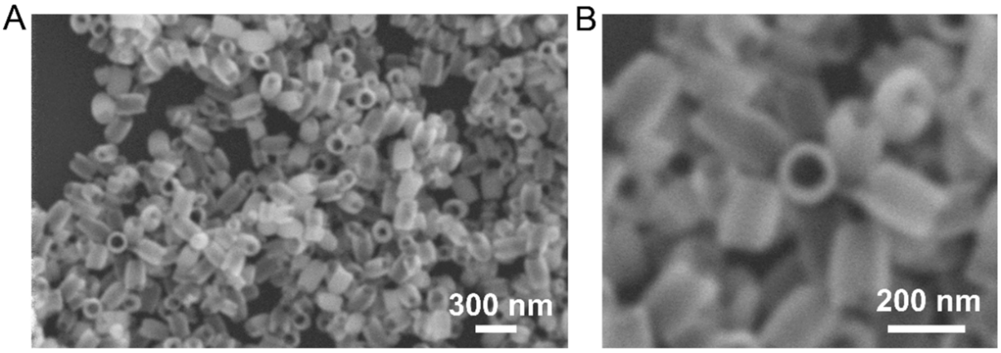

Figure S1. SEM images of α−Fe_2O_3 nanotubes obtained from the hydrothermal procedure at (**A**) low and (**B**) high magnification.

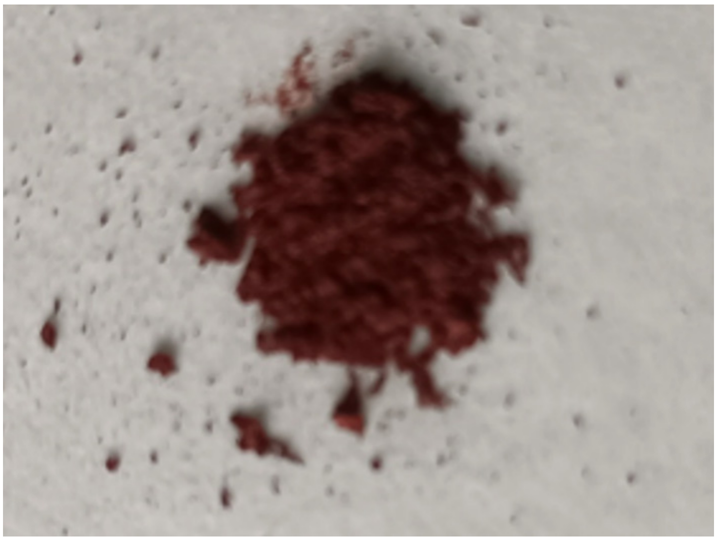

Figure S2. The digital photograph of Fe_2O_3 TNMs.

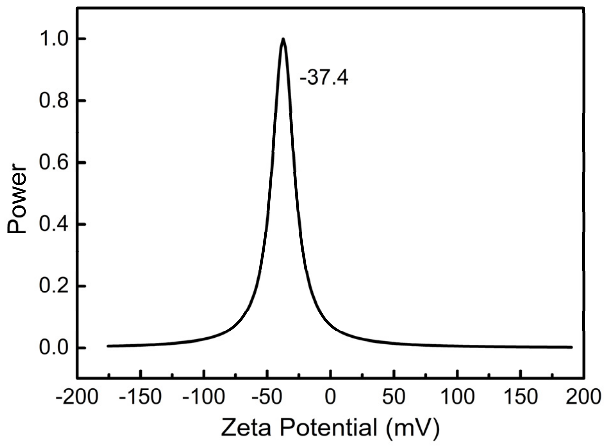

Figure S3. Zeta potential of the Fe_2O_3 TNMs.

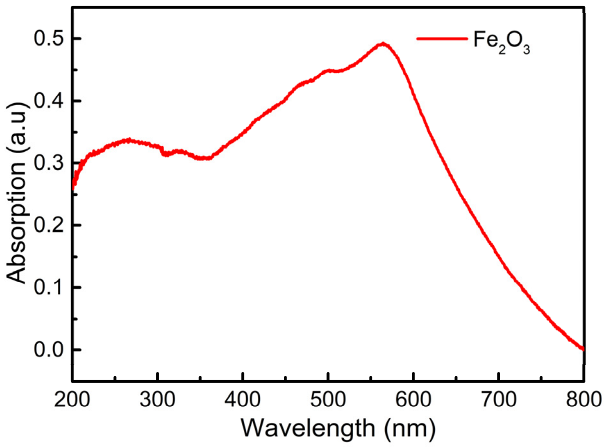

Figure S4. The UV−vis absorbance spectrum of the Fe_2O_3 TNMs.

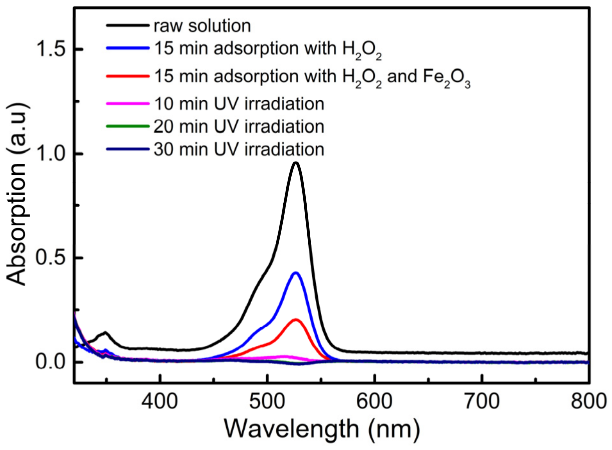

Figure S5. Absorbance spectra of Rhodamine 6G in the medium with the Fe_2O_3 TNMs before and after UV exposure (1.8 W/cm^{-2}) for different times.

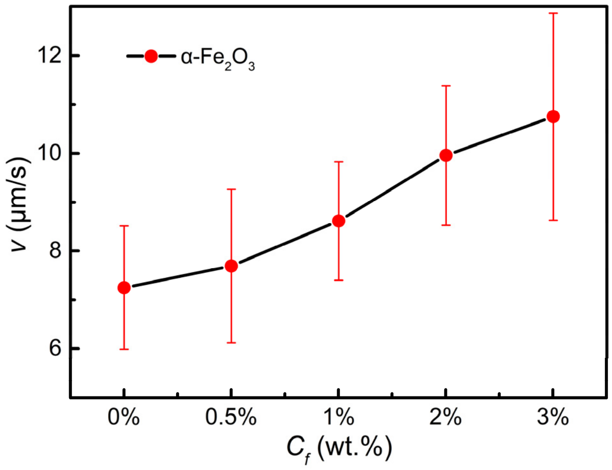

Figure S6. Speed (v) of the α−Fe_2O_3 nanotubes as a function of C_f. The light intensity (I) is kept at 513 mW/cm^2.

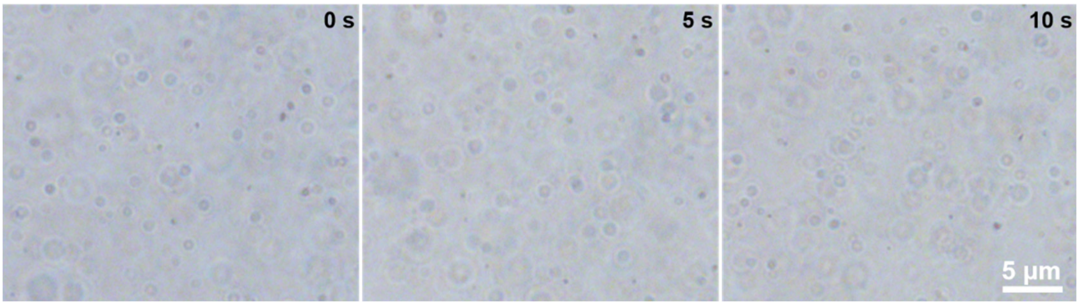

Figure S7. Time−lapse microscopic images of the Fe_2O_3 TNMs in pure water under UV_Z irradiation (I = 513 mW/cm^2).

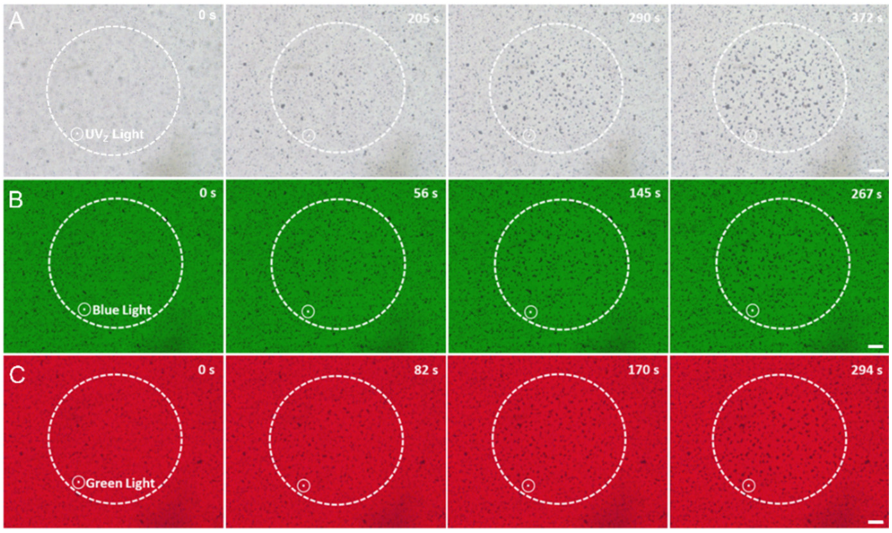

Figure S8. Clustering behaviors of the Fe_2O_3 TNMs in a circular spot of (**A**) UV_Z (360 nm), (**B**) blue (480 nm), and (**C**) green light (538 nm), respectively. Scale bars are 20 μm.

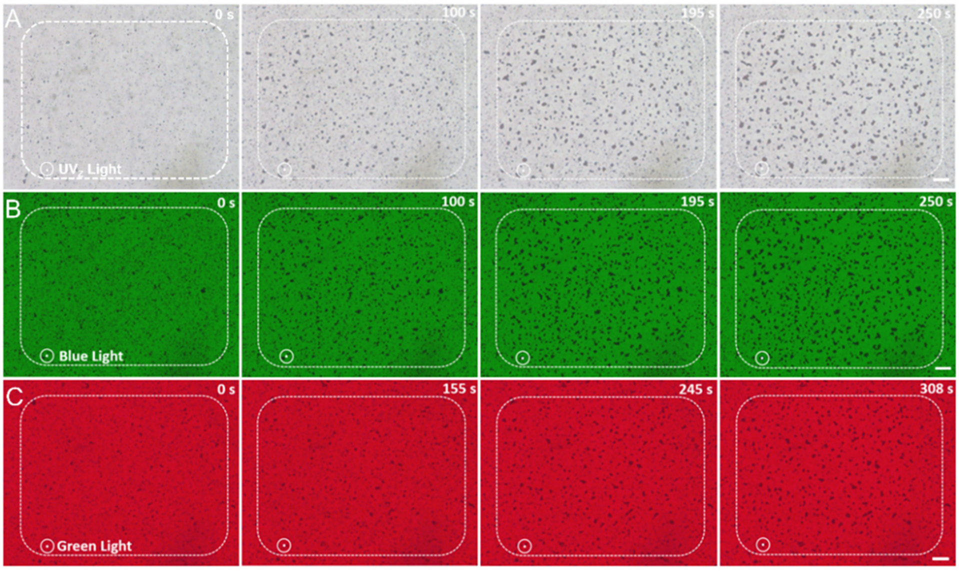

Figure S9. Clustering behaviors of the Fe_2O_3 TNMs in a rectangular spot of (**A**) UV$_Z$ (360 nm), (**B**) blue (480 nm), and (**C**) green light (538 nm), respectively. Scale bars are 20 μm.

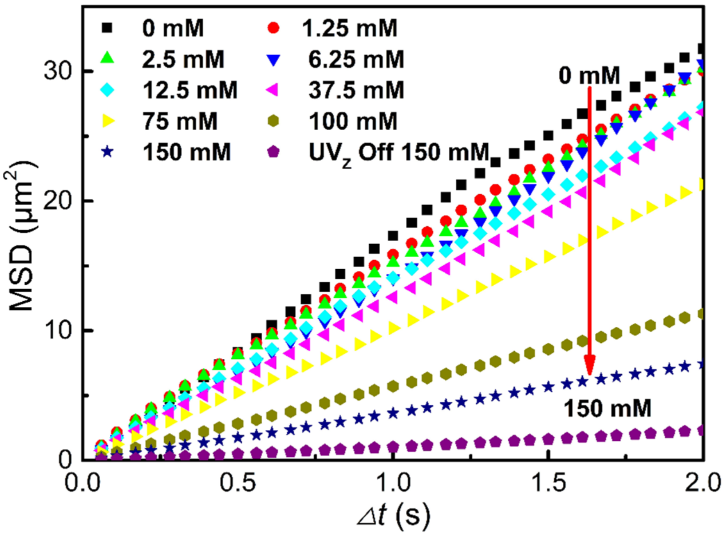

Figure S10. Average MSD of the Fe_2O_3 TNMs at different C_e versus the time interval (Δt).

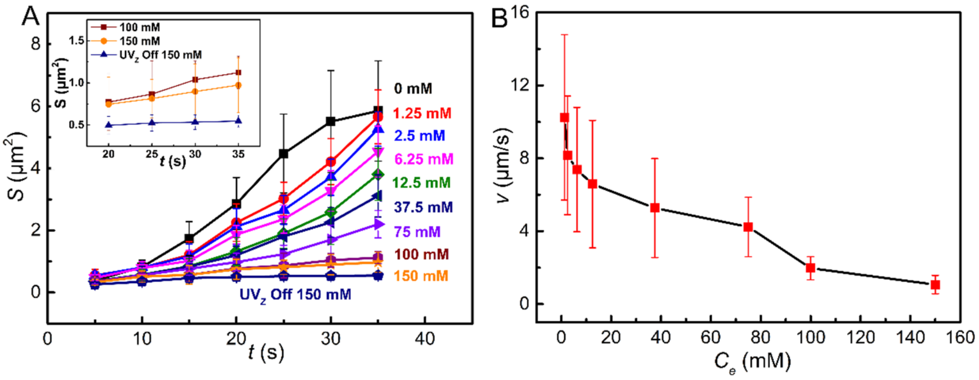

Figure S11. (**A**) The increment of cluster size (S) over time (t) in the medium with different C_e. (**B**) The speed (v) of the formed clusters at different C_e.

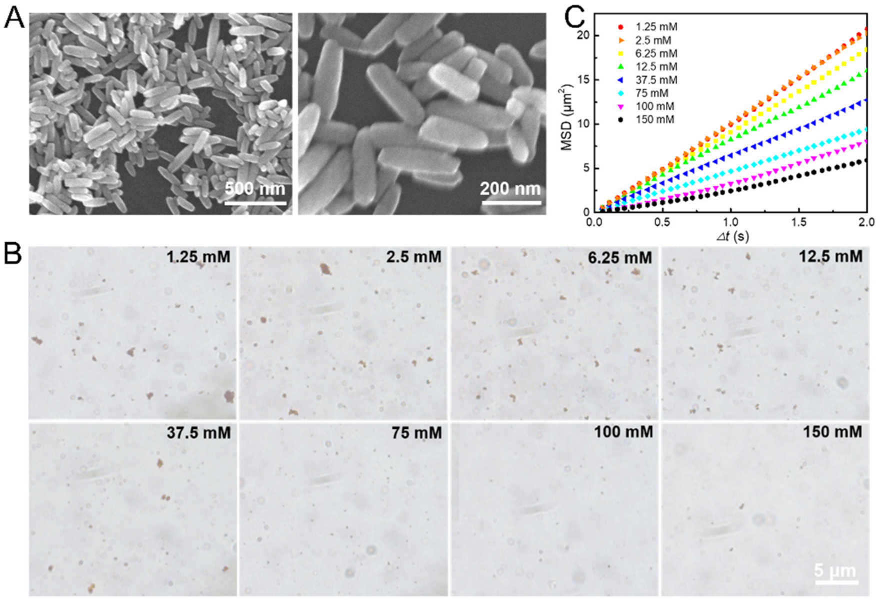

Figure S12. Characterization and ion−tolerant motion behaviors of solid Fe_2O_3 nanomotors. (**A**) SEM images of solid Fe_2O_3 nanomotors at low and high magnification. (**B**) Microscopic images describing the clustering of solid Fe_2O_3 nanomotors after irradiated by UV_Z irradiation for 30 s. (**C**) Average MSD of the solid Fe_2O_3 nanomotors at different C_e versus the time interval (Δt). The I of the applied UV_Z light in these experiments is 513 mW/cm^2.
